# Bliss & Sharp

**Published:** 1868-03-01

**Authors:** 


### *Messrs. Bliss & Sharp,*

144 Lake Street, have shown us a neat little *tubular cork-screw*, which seems well adapted for its purpose.

"By screwing the tap into the cork of a bottle containing soda- water champagne, or other ærated beverage, any quantity may be drawn off at pleasure, and what remains in the bottle will not lose its effervescing property. In this manner the contents of the bottle can be taken at pleasure, without extracting the cork, and all waste avoided.

Its convenience is so obvious, that we presume it will find its way into general use. The following directions are given:

"The wire must not be unloosed. Make room between the double wires on the top of the cork, by setting them aside so that the tap shall enter between them; then with one of the loose points on the end of the tap, screw it through the centre of the cork till the point of the screw is seen under the cork. Should the pressure prevent the point from dropping into the bottle, give it a shake, and the point will fall away. Hold the bottom of the bottle upwards, turn the stopper, and draw the quantity required. In this manner ten or more draughts may be had from one bottle. When the bottle is empty draw the cork out."

